# Social isolation, inflammation, and cancer mortality from the National Health and Nutrition Examination Survey - a study of 3,360 women

**DOI:** 10.1186/s12889-021-11352-0

**Published:** 2021-07-02

**Authors:** Alexander Koh-Bell, Joshua Chan, Amandeep K. Mann, Daniel S. Kapp

**Affiliations:** 1 Brown University, Providence, RI USA; 2 Stanford University, Stanford, CA USA; 3 Palo Alto Medical Foundation Research Institute, 795 El Camino Real, Palo Alto, CA 94301 USA; 4 Department of Radiation Oncology, Stanford University School of Medicine, Stanford, CA USA

**Keywords:** Social network index, Cancer mortality, C-reactive protein, Fibrinogen

## Abstract

**Background:**

This study evaluates the role of social isolation on inflammation and cancer mortality among women.

**Methods:**

Data were abstracted from the U.S. National Health and Nutrition Examination Survey from 1988 to 1994. The Social Network Index was used to assess participants’ degree of social isolation. C-reactive protein and fibrinogen levels were included as markers of inflammation. We used the National Death Index to identify causes and dates of mortality. Chi-square and multivariable Cox regressions were employed for statistical analyses.

**Results:**

Of 3360 women (median age: 54 years), the most isolated, very isolated, somewhat isolated, and not isolated comprised 14.5, 30.2, 37.1, and 18.2% of the sample, respectively. The most isolated participants were more likely to have low income (56.8% vs 12.2%, *p* < 0.001), have fewer years of education (40.8% vs 12.3%; *p* < 0.001), have low physical activity (27.3% vs 14.7%; *p* < 0.003), be obese (32.5% vs 24.4%; *p* = 0.02), and be current smokers (34.2% vs 10.3%; *p* < 0.001) compared to the not isolated ones. Mean fibrinogen levels increased with degree of social isolation (*p* = 0.003), but C-reactive protein showed no association (*p* = 0.52). Kaplan-Meier estimates indicated higher cancer mortality rates among participants with elevated fibrinogen levels, though not with statistical significance (*p* = 0.08). Furthermore, there was no association between social isolation and cancer mortality (*p* = 0.54). On multivariate analysis, obesity (HR = 1.56; 95% CI: 1.11–2.18), higher education (HR = 1.36; 95% CI: 1.01–1.83), and smoking (HR = 4.42, 95% CI: 2.84–6.88) were independent predictors for cancer mortality, while high physical activity predicted for lower mortality from cancer (HR = 0.67, 95% CI: 0.51–0.87). However, social isolation was not a predictor.

**Conclusion:**

Social isolation among women was associated with an increased level of fibrinogen, but not associated with cancer mortality. The relationship between inflammation and cancer mortality warrants further investigation.

**Supplementary Information:**

The online version contains supplementary material available at 10.1186/s12889-021-11352-0.

## Background

Social isolation, defined as a lack of interpersonal interaction, community engagement, and personal relationships, has been shown to be associated with higher inflammation as well as higher risk for mortality [1, 2]. After controlling for various mortality risk factors, several epidemiological studies have shown that the inflammatory biomarker C-reactive protein (CRP) was higher among socially isolated individuals [2, 3]. On the other hand, a study of Swedish men did not find a similar association between the inflammatory marker fibrinogen and social isolation [4]. Evaluating the relationship between cancer mortality and social isolation, multiple reports have shown increased cancer mortality in socially isolated individuals, but these results have differed between men and women [5–9].

Differences between men and women in inflammatory response may also contribute to the variation in these reports across sex [10]. Researchers have reported on the association of social isolation, inflammation, and mortality and found that social isolation was significantly correlated with cancer mortality in men, but not women [6]. In contrast, Marcus et al. found this association between social isolation and cancer mortality to only hold for women [11]. However, this report did not adjust for inflammation or other health-related factors such as body mass index (BMI) and physical activity. While there is literature supporting the relationships between isolation and inflammation as well as inflammation and cancer, there are few multidisciplinary reports that assess the associations of these three topics [12].

Previous reports have indicated that inflammation may be a mediator in the relationship between social isolation and cancer mortality [6]. C-reactive protein (CRP) and fibrinogen are acute-phase proteins and markers of inflammation associated with cancer and cardiovascular disease. Studies indicate an association between CRP and cancer mortality, particularly for colorectal cancer [13]. The inflammatory marker fibrinogen has been shown to be associated with risk and prognosis of ovarian and other cancers [14, 15].

Due to conflicting reports on how men and women’s social isolation relates to inflammation and cancer mortality, we chose to analyze these relationships further, specifically among women. In particular, we evaluate the relationship between social isolation and inflammatory biomarkers as well as the association between social isolation and cancer mortality among women. In this report, we aim to clarify these relationships by analyzing baseline participant data along with rates of cancer mortality in a nationally representative sample of 3360 women from the US National Health and Nutrition Examination Survey (NHANES).

## Methods

### Data source

Our cohort consists of women of age 40 or above from the US National Health and Nutrition Examination Survey database from 1988 to 1994 (NHANES III). Due to the database’s inclusion of fibrinogen data only among participants of age 40 and older, no younger participants were included in our cohort. NHANES is a cross-sectional examination survey conducted by the Centers for Disease Control and Prevention to evaluate the health and nutrition of the U.S. population [16]. This database includes information on demographics, health, and nutrition based on home interviews and physical examinations conducted in mobile examination centers (MEC). Participants who were 60 years and older, Mexican American, and Non-Hispanic Black were oversampled in the NHANES III database.

### Demographics and behavioral characteristics

From this dataset, we abstracted age, race, income level, and education as our demographic factors, as well as BMI, self-reported health status, health conditions commonly linked to inflammation such as diabetes and cardiovascular disease (CVD), smoking, alcohol, non-steroidal anti-inflammatory drugs (NSAIDs), and physical activity. Age was dichotomized using the median age (54 years) of the overall sample and race was categorized as White, Black, Mexican American, or other. Education level was assessed by the highest grade or year of school that participants had completed. Additionally, participants’ income level was determined by poverty income ratio (PIR), the ratio of family income to the poverty threshold adjusted by economic inflation. As defined by the U.S. Census, income levels are categorized as poverty: PIR < 1, low income: 1.0 ≤ PIR < 2.0, middle income: 2.0 ≤ PIR < 4.0, and high income: PIR ≥ 4.0. We have elected to include participants who were in poverty as members of our low-income group. Body mass index (BMI) was measured in the MEC and was categorized as obese for BMI of 30 and above, based on the Centers for Disease Control and Prevention categorization. Self-reported health status was assessed by participant responses to the question, “Would you say your health in general is excellent, very good, good, fair, or poor?” Additionally, participants with diabetes and cardiovascular disease were assessed by a self-reported questionnaire on their previous diagnosis by their doctor. Smoking status was self-reported by participants and defined as nonsmoker, former, or current smoker. We also investigated the number of packs of cigarettes smoked per day among the current smokers and dichotomized it by the median value (1 pack/day). Alcohol status of participants was classified as either nondrinker, former, or current drinker. We then dichotomized current drinkers by the median value of 2 drinks/day. Consumption of NSAIDs such as aspirin, acetaminophen, and ibuprofen was also assessed. Participants who were taking 30 or more pills/doses in the past month were considered regular users. Finally, physical activity was determined by how participants compare their physical activity to their peers.

### Social network index

Social Network Index (SNI) is a measure of social isolation created by Berkman and Syme that evaluates individuals’ marriage or partnership, support from friends or family, and religious or other group participation [1]. An individual’s SNI is defined by the number of the following criteria that they meet: married or living with a partner, having on average greater than 155 contacts or social interactions with family, friends, or neighbors per year, attending religious services more than 3 times per year, and involved in any club or organization such as a religious, fraternal, school, or athletic group. Home interviews took place with questions about these factors and answers were combined into a Social Network Index metric on a scale of 0 to 4 (0 being the highest level of isolation) [17]. We elected to group together individuals with a SNI of 0 or 1 and categorize them as ‘Most Isolated’.

### Cancer mortality and exclusion criteria

We obtained data on causes and dates of mortality from the National Death Index (NDI) and used the International Classification of Diseases codes to determine underlying causes of death (C00-C97). From the crude sample of females from NHANES III (*N* = 17,299), we limited our data to participants who were evaluated in home interviews and mobile examination centers (*N* = 16,037) and were eligible for mortality follow-up (*N* = 9176). We elected to solely study women 40 years and older (*N* = 5073) because NHANES lacked fibrinogen data for participants < 40 years old. Then, we only included participants with comprehensive information on fibrinogen, CRP, and social isolation indices (*N* = 4556). Only participants with baseline CRP measurements below 10 mg/dL were included in our study (*N* = 4448) because CRP levels ≥10 mg/dL are often associated with influenza [11]. In addition, we excluded 638 participants who had a history of cancer and 2 participants with missing information on their history of cancer. Because NHANES reported participants who are 90 years and above as “90” we only included those who were < 90 years old (*N* = 3980). Additionally, we excluded participants with missing information on body mass index, self-reported health status, physical activity, education, poverty-income ratio, alcohol, and NSAIDs. The final analytic sample consisted of 3360 participants after incorporating the above exclusion criteria.

### Laboratory analyses of inflammatory markers

We analyzed NHANES measurements of two serum inflammatory biomarkers, C-reactive protein (CRP) and fibrinogen. These measurements were made from assessment of blood samples collected from NHANES participants. CRP levels were examined using latex-enhanced nephelometry, an analysis of light scattering of antigen-antibody complexes with latex particles. Fibrinogen levels were measured through thrombin clotting time, in which thrombin was used to enzymatically convert fibrinogen into fibrin [18]. In our analysis, CRP was categorized as a categorical variable. Because the majority (61%) of participants in our study had CRP levels below the detection limit (< 0.22 mg/dL), these participants were categorized as one group. Others with CRP levels at or above the detection level were dichotomized using a median value (0.63 mg/dL). Fibrinogen was treated as a continuous variable for the univariate and multivariate analysis. However, on Kaplan-Meier curves we categorized fibrinogen levels as < 200 mg/dL, 200–400 mg/dL, and > 400 mg/dL.

### Statistical analysis

Chi-square tests and t-tests were used to determine the associations between social isolation and all other collected baseline and demographic variables. To determine whether social isolation or any other factor was independently associated with cancer mortality, we performed multivariate analyses using adjusted Cox-proportional hazard models. In addition, multistage stratified, clustered probability, and sampling weights of the U.S. population were included in our analysis. Data analyses were conducted using SAS® Enterprise Guide 7.1 (SAS Institute Inc., Cary, NC, USA). This study was exempted from the IRB approval because it utilized a public-use data file and did not contain identifying information of the participants.

## Results

Of 3360 women (median age: 54 years; range: 40–89), 81.4% were White, 8.8% were Black, 3.1% were Mexican American, and 6.6% were of another race. Low, middle, and high-income levels were represented in 31.5, 38.0, and 30.5% of participants, respectively. 73.8% of participants had completed high school or the 12th year of education, while the remaining 26.2% had not. Additionally, 27.0% of participants were obese. The sample included 20.0% participants who currently smoke tobacco, 26.1% who formerly smoked, and 54.0% who never smoked. Relative physical activity was divided into less active, about the same, and more active at 19.9, 43.9, and 36.2%, respectively (Table 1).

**Table 1:** Associated Characteristics by Social Network Index

Characteristics	Overall	0/1 = Most Isolated	2 = Very Isolated	3 = Somewhat Isolated	4 = Not Isolated	***P***-value
**Overall N (%)**	*N* = 3360	543 (14.5%)	1134 (30.2%)	1198 (37.1%)	485 (18.2%)	
**Age**						0.90^a^
Median (range)	54 (40–89)	54 (40–89)	55 (40–89)	54 (40–89)	54 (40–88)	
Younger than 54	46.2%	46.6%	44.7%	46.7%	47.4%	
54 years and older	53.8%	53.3%	55.3%	53.3%	52.6%	
**Race/Ethnicity**						< 0.001^a^
White	81.4%	75.4%	78.9%	81.7%	89.8%	
Black	8.8%	9.4%	10.6%	8.6%	5.9%	
Mexican American	3.1%	3.7%	3.7%	3.1%	1.5%	
Other^b^	6.6%	11.5%	6.7%	6.6%	2.7%	
**Income Level**						< 0.001^a^
Low income	31.5%	56.8%	38.0%	25.8%	12.2%	
Middle income	38.0%	30.6%	37.0%	38.1%	45.5%	
High income	30.5%	12.6%	24.9%	36.1%	42.3%	
**Education**						< 0.001^a^
Fewer than 12 years	26.2%	40.8%	32.9%	21.7%	12.3%	
12 years or more	73.8%	59.2%	67.1%	78.3%	87.7%	
**BMI (kg/m**^**2**^**)**						0.02^a^
< 30.0 kg/m^2^	73.0%	67.5%	70.6%	75.7%	75.6%	
≥ 30.0 kg/m^2^	27.0%	32.5%	29.4%	24.3%	24.4%	
**Diabetes Diagnosis**						0.20^a^
No	92.1%	89.9%	91.2%	93.1%	93.6%	
Yes	7.9%	10.1%	8.8%	6.9%	6.4%	
**Cardiovascular Disease Diagnosis**						0.02^a^
No	64.0%	59.0%	60.8%	66.7%	68.0%	
Yes	36.0%	41.0%	39.2%	33.3%	32.0%	
**Smoking Status**						< 0.001^a^
Nonsmoker	54.0%	38.0%	46.8%	62.0%	62.4%	
Former	26.1%	27.8%	27.6%	23.5%	27.4%	
Current	20.0%	34.2%	25.6%	14.5%	10.3%	
**Smoking Amount**						< 0.001^a^
Nonsmoker	54.0%	38.0%	46.8%	62.0%	62.4%	
Former	26.1%	27.8%	27.6%	23.5%	27.4%	
Current						
< 1 pack/day	8.9%	15.5%	10.7%	6.6%	5.1%	
≥ 1 pack/day	11.1%	18.7%	14.9%	7.9%	5.2%	
**Alcohol Status**						0.24^a^
Nondrinker	21.8%	19.2%	20.8%	24.3%	20.3%	
Former	41.0%	44.6%	44.1%	37.8%	39.6%	
Current	37.2%	36.2%	35.1%	37.9%	40.1%	
**Alcohol Consumption**						< 0.001^a^
Nondrinker	21.8%	19.2%	20.8%	24.3%	20.3%	
Former	41.0%	44.6%	44.1%	37.8%	39.6%	
Current						
1–2 drinks /day	30.4%	21.7%	28.2%	32.2%	37.3%	
≥ 3 drinks/day	6.8%	14.6%	6.9%	5.7%	2.8%	
**Relative Physical Activity**						0.003^a^
About the Same	43.9%	37.0%	45.0%	43.9%	47.5%	
Less Active	19.9%	27.3%	22.4%	17.4%	14.7%	
Most Active	36.2%	35.6%	32.6%	38.6%	37.8%	
**Self-Reported Health Status**						0.001^a^
Excellent	32.5%	23.8%	30.0%	35.8%	36.6%	
Very Good	27.7%	29.6%	27.2%	26.6%	29.4%	
Good	29.2%	28.7%	32.0%	27.7%	27.9%	
Fair	9.1%	14.4%	9.1%	8.6%	5.8%	
Poor	1.6%	3.4%	1.7%	1.3%	0.3%	
**Non-Steroidal Anti-Inflammatory Drug Use**						0.02^a^
Nonuser^c^	21.1%	26.8%	22.0%	20.2%	17.0%	
User	78.9%	73.2%	78.0%	79.8%	83.0%	
**C-Reactive Protein (mg/dL)**						0.52^a^
≤ 0.21 mg/dL	61.0%	60.1%	59.6%	61.3%	63.1%	
0.22–0.63 mg/dL	18.7%	20.4%	17.6%	18.2%	20.2%	
> 0.63 mg/dL	20.3%	19.5%	22.8%	20.5%	16.7%	
**Fibrinogen (mg/dL)**	308	318	312	309	289	0.003^d^

Data are % unless otherwise specified

Note: percentages may not add up to 100 due to rounding

^a^ Chi-square test was performed to calculate *p*-values between the two categorical variables

^b^ Other race consists of mixed race, Asians, and any other race not specified in the NHANES III database

^c^ Non-steroidal anti-inflammatory drugs (NSAID) nonuser includes those who never taken NSAID and never taken NSAID on a regular basis (< 30 pills/month)

^d^ T-test was used to calculate *p*-values for the mean differences of fibrinogen levels between the social network index groups

With respect to Social Network Index, 14.5, 30.2, 37.1, and 18.2% were most isolated, very isolated, somewhat isolated, and not isolated, respectively. Significant differences were found between isolated and non-isolated participants in demographic and other baseline variables. Members of the most isolated group were more likely than that of the not isolated group to be of low income (56.8% vs 12.2%, *p* < 0.001), have fewer than 12 years of education (40.8% vs 12.3%, *p* < 0.001), be less physically active (27.3% vs 14.6%, *p* = 0.003), be obese (32.5% vs 24.4%, *p* = 0.02) and currently smoke (34.2% vs 10.3%, *p* < 0.001). Of current smokers, the most isolated were more likely than the not isolated participants to smoke an average of one or more pack per day (18.7% vs 5.2%, *p* < 0.001), and of current drinkers, the most isolated were more likely than the not isolated to consume at least three drinks per day (14.6% vs 2.8%, *p* < 0.001). (Supplemental Table 1) The most isolated women were also more likely than the not isolated women to have cardiovascular disease (41.0% vs 32.0%, *p* = 0.02) and to have never been regular NSAID users (26.8% vs 17.0%, *p* = 0.02), relative to the not isolated group (Table 1). The *p*-values refer to comparisons across the rows of Table 1.

**Fig. 1:**
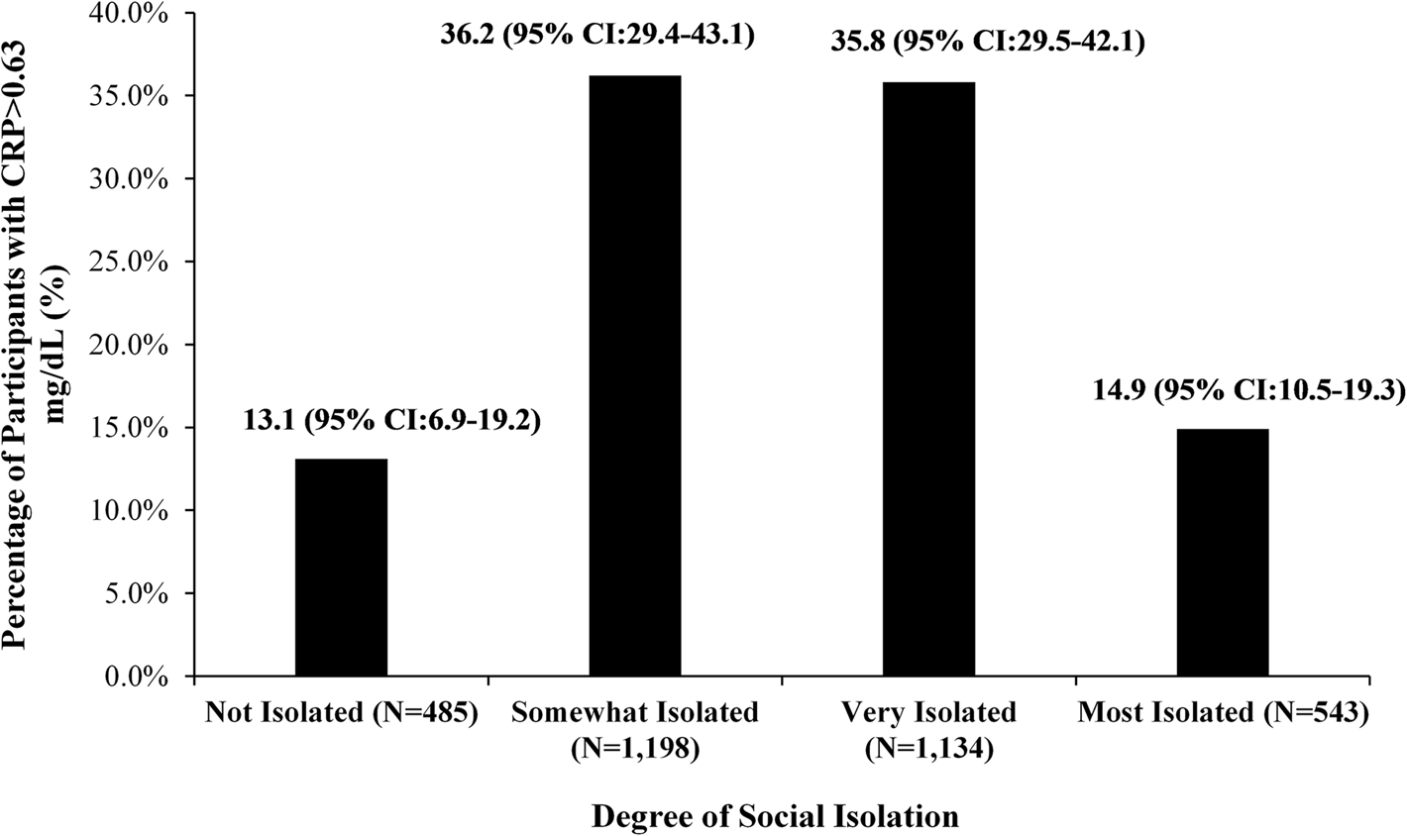
Association between Social Network Index and Elevated C-Reactive Protein (Chi-squared *p* = 0.52)

**Fig. 2:**
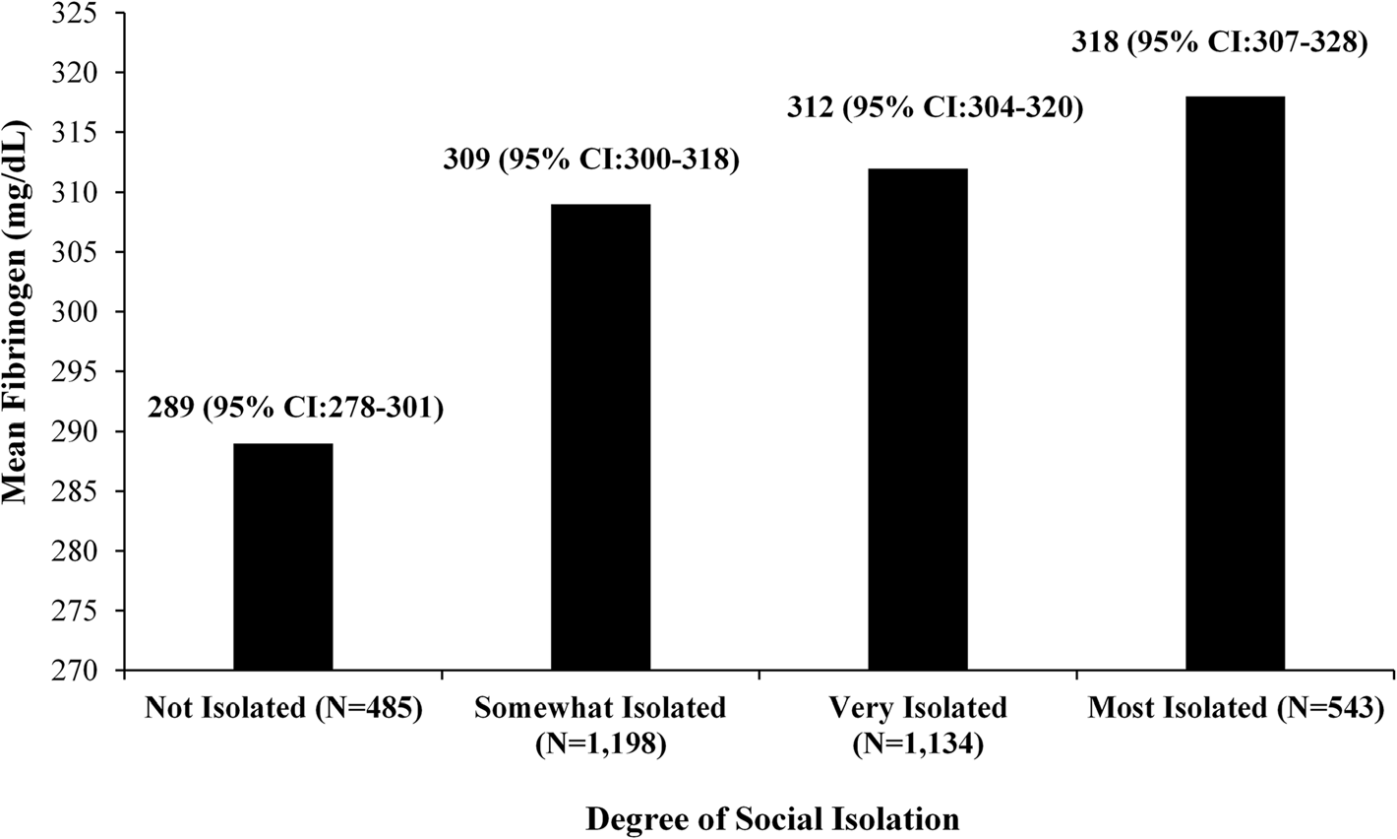
Association between Social Network Index and Fibrinogen (T-test *p* = 0.002)

We evaluated the associations between social isolation and both CRP and fibrinogen levels through Chi-square analyses. No significant association was found between social isolation and CRP level (Fig. 1). However, fibrinogen levels consistently increased across Social Network Index categories from least to most isolated, with mean fibrinogen measurements of 289 mg/dL, 309 mg/dL, 312 mg/dL, and 318 mg/dL, respectively (*p* = 0.003) (Fig. 2) (Table 1).

**Fig. 3:**
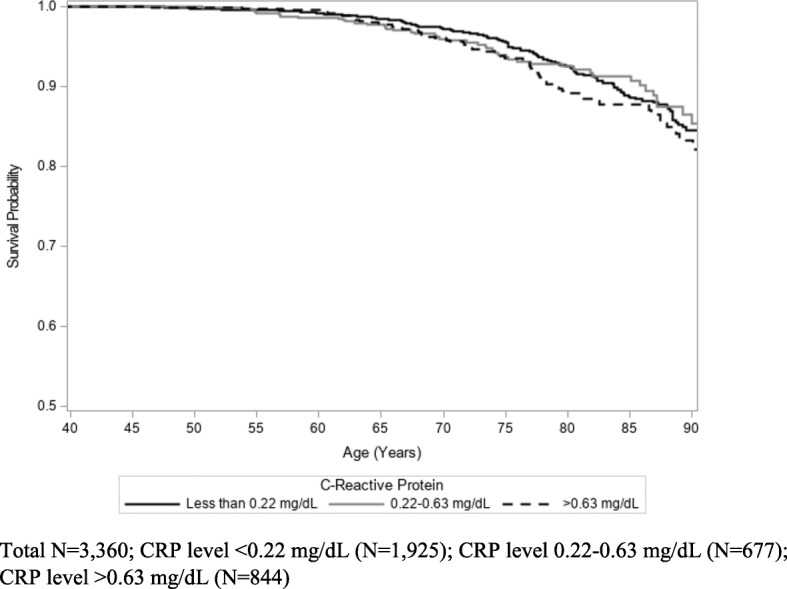
Kaplan-Meier Estimates of Cancer Mortality by Baseline Level of C-Reactive Protein (log-rank *p* = 0.55)

**Fig. 4:**
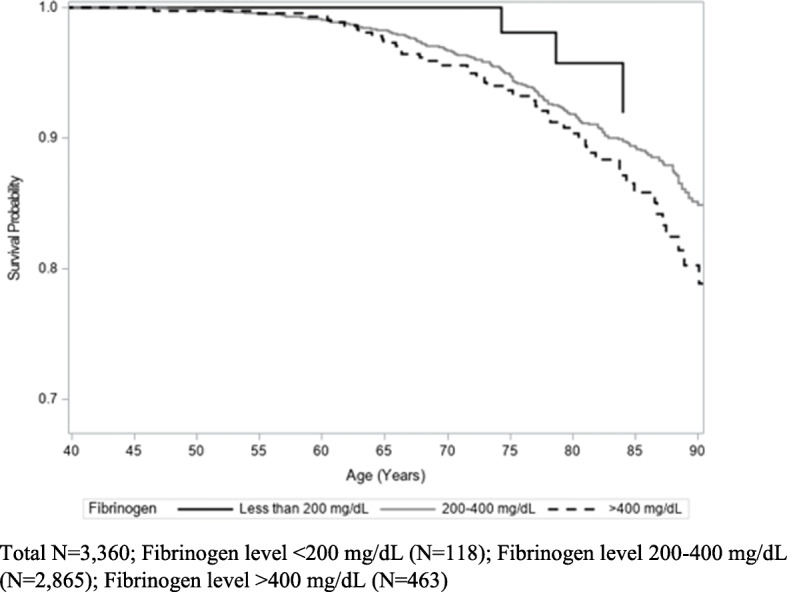
Kaplan-Meier Estimates of Cancer Mortality by Baseline Level of Fibrinogen (log-rank *p* = 0.08)

**Fig. 5:**
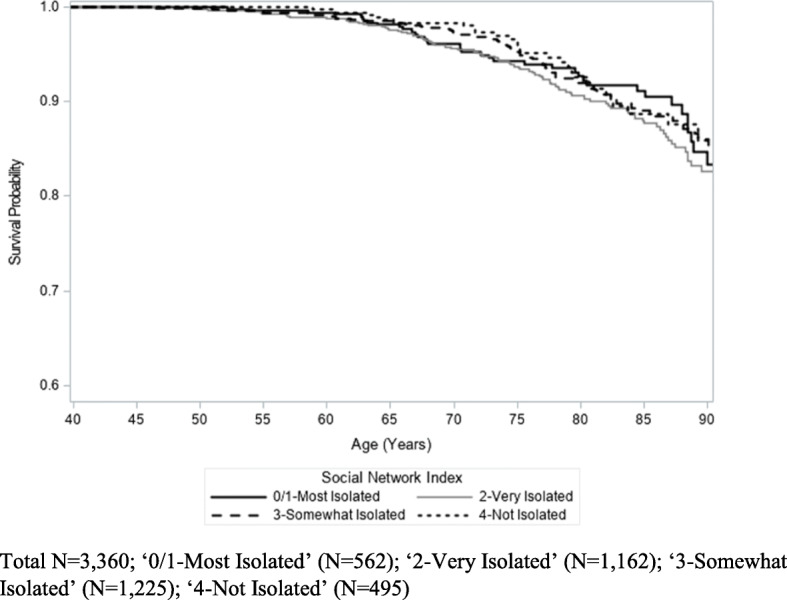
Kaplan-Meier Estimates of Cancer Mortality by Social Network Index (log-rank *p* = 0.54)

Kaplan-Meier analysis did not show a relationship between CRP levels and cancer mortality (*p* = 0.55) (Fig. 3). While not statistically significant, participants with high fibrinogen measurements (> 400 mg/dL) appeared to have higher cancer mortality rates than those with lower fibrinogen measurements (< 200 mg/dL and 200–400 mg/dL) (*p* = 0.08) (Fig. 4). In addition, there was no association between degree of social isolation and cancer mortality (*p* = 0.54) (Fig. 5).

On multivariate analysis, obesity (HR = 1.56; 95% CI: 1.11–2.18), higher education (HR = 1.36; 95% CI: 1.01–1.83), and current smoking (HR = 4.42, 95% CI: 2.84–6.88) were independent predictors for cancer mortality, while high physical activity independently predicted for lower cancer mortality (HR = 0.67; 95% CI: 0.51–0.87). However, social isolation was not independently associated with cancer mortality after adjusting for demographic, socioeconomic and behavioral factors (HR = 0.76; 95% CI: 0.28–2.06). In addition, both elevated CRP (HR = 0.88; 95% CI: 0.53–1.45) and fibrinogen (HR = 1.001; 95% CI: 1.00–1.003) levels were not independently associated with cancer mortality (Table 2).

**Table 2:** Multivariate Cox Proportional Hazard Model of Associated Factors with Cancer Mortality

Characteristics	Hazard Ratio	95% Confidence Interval	***P***-value
**Social Network Index**			
4 = Not isolated	1		
3 = Somewhat isolated	0.72	0.41–1.27	0.25
2 = Very isolated	0.81	0.39–1.70	0.58
0/1 = Most isolated	0.76	0.28–2.06	0.58
**Race/Ethnicity**			
White	1		
Black	1.35	0.84–2.17	0.22
Mexican American	1.13	0.70–1.82	0.62
Other^a^	1.04	0.22–4.82	0.96
**Income Level**			
Low income	1		
Middle income	0.89	0.57–1.38	0.60
High income	0.98	0.60–1.60	0.94
**Education**			
Fewer than 12 years	1		
12 years or more	1.36	1.01–1.83	0.04
**BMI (kg/m**^**2**^**)**			
< 30.0 kg/m^2^	1		
≥ 30.0 kg/m^2^	1.56	1.11–2.18	0.01
**Diabetes Diagnosis**			
No	1		
Yes	0.72	0.33–1.55	0.39
**Cardiovascular Disease Diagnosis**			
No	1		
Yes	0.78	0.49–1.24	0.29
**Smoking Status**			
Nonsmoker	1		
Former	2.07	1.15–3.73	0.02
Current	4.42	2.84–6.88	< 0.001
**Alcohol Consumption**			
Nondrinker	1		
Former	1.15	0.72–1.83	0.56
Current	0.96	0.57–1.65	0.89
**Relative Physical Activity**			
About the same	1		
Less active	1.57	1.10–2.26	0.01
Most active	0.67	0.51–0.87	0.004
**Self-reported Health Status**			
Excellent	1		
Very good	1.47	0.98–2.20	0.06
Good	1.37	0.97–1.94	0.07
Fair	1.05	0.62–1.77	0.85
Poor	0.41	0.14–1.19	0.10
**Non-Steroidal Anti-Inflammatory Drug Use**			
Nonuser^b^	1		
User	1.13	0.79–1.60	0.49
**C-Reactive Protein (mg/dL)**			
≤ 0.21 mg/dL	1		
0.22–0.63 mg/dL	1.19	0.80–1.77	0.37
> 0.63 mg/dL	0.88	0.53–1.45	0.60
**Fibrinogen (mg/dL)**	1.001	1.00–1.003	0.49

^a^ Other race consists of mixed race, Asians, and any other race not specified in the NHANES III database

^b^ Non-steroidal anti-inflammatory drugs (NSAID) nonuser includes those who never taken NSAID and never taken NSAID on a regular basis (< 30 pills/month)

## Discussion

Social isolation has been shown to be associated with inflammatory biomarker levels [1, 2]. Moreover, social isolation may lead to increased risk for cancer mortality [11]. However, the impact of social isolation in women may differ from that in men, particularly in relation to inflammatory response and cancer mortality [6–9, 13]. In our analysis of 3360 adult females based on a large, nationally representative survey, we found that socially isolated participants were more likely to have lower socioeconomic status, lower rates of physical activity, and higher rates of obesity and smoking. Lack of social connectedness was also associated with heightened levels of serum fibrinogen, but not CRP. This association between social isolation and fibrinogen but not CRP raises questions regarding the biomarker-specific effects of isolation. While social isolation and inflammatory biomarkers have both been associated with cancer mortality in existing studies, both CRP and social isolation showed no association with cancer mortality on our Kaplan-Meier estimates [11, 13, 14]. Although there may be a clinically relevant survival difference between participants based on fibrinogen levels, this was not statistically significant. Furthermore, there was no significant relationship between social isolation and cancer mortality after adjusting for demographic, socioeconomic, and behavioral risk factors. While our analysis did not find an association between inflammatory biomarkers and cancer mortality or between social isolation and cancer mortality, relationships between these factors may still exist. These associations may have not been observed in the current report due to our methods, sampling, and limited cohort size, or may instead reflect a true lack of association in the general population.

In our study, we found a direct association between the degree of social isolation and fibrinogen levels. Similarly, the Framingham Offspring Study group by Kim et al. found elevated fibrinogen levels in those with less social connection [12]. In addition, Nersesian et al. found high fibrinogen levels to be associated with loneliness and lack of social connection in their study of middle-aged men and women [19]. Prior studies have investigated the potential mechanisms underlying the relationship between social isolation and inflammation. This relationship may be mediated by increased psychological stress in isolated individuals, which is correlated with heightened inflammation [20–22]. Loneliness may activate the sympathetic nervous system and Hypothalamic Pituitary Adrenocortical (HPA) axis, both of which are critical components to the body’s stress response [23, 24]. Furthermore, dysregulation of the HPA-axis may be associated with chronic stress and subsequently higher inflammation levels [25]. Researchers have also suggested that social isolation may result in heightened inflammatory sensitivity to biological and social stressors, resulting in elevated inflammation among isolated individuals [26].

Conversely, it is also possible that inflammation can induce social withdrawal. Raison and Miller suggest that inflammatory cytokines activate conservation and withdrawal behavior through the basal ganglia [27]. More specifically, inflammatory cytokines induce a “sickness behavior,” including social withdrawal, that provide an adaptive defense mechanism to protect against infection by avoiding exposures and conserving energy [28]. Furthermore, the expression of the neuropeptide Y gene has been shown to be correlated with inflammation as well as social isolation and major depressive disorder [29–31].

Prior studies have shown an association between CRP and social integration [21, 32]. In a New England regional study of over 2000 adults, the most socially isolated individuals were more than twice as likely to have elevated CRP levels compared to the most socially integrated participants [33]. However, these studies evaluated social isolation and CRP among both men and women. Aligning with our findings, prior studies have not shown a significant association between social isolation and CRP levels solely in women [2, 3]. Further studies are warranted to investigate the biological role of sex in the relationship between isolation and CRP level.

In this current report, elevated fibrinogen levels appeared to be associated with higher cancer mortality among women, though this association was not statistically significant. However, on multivariate analysis, both CRP and fibrinogen were not independent predictors for cancer mortality. A meta-analysis by Ni et al. found a higher relative risk for cancer mortality among participants with high CRP levels; however, this study did not limit participants to solely women [34]. Similar to our findings, Wulaningsih et al. was unable to demonstrate a significant association between CRP levels and cancer mortality in women using the NHANES III sample, but the authors did find this relationship in their male cohort [35]. In an NHANES III study of over 10,000 women, Gathirua-Mwangi and colleagues also did not find an association between CRP and cancer mortality [36]. Furthermore, Li et al. found that elevated high-sensitivity C-reactive protein levels were associated with higher cancer mortality among men, but this relationship was insignificant among women [37]. The sex differences from these studies may be explained by the reported lower levels of cortisol levels in women in response to stress compared to men [11, 38, 39]. Kudielka and Kirschbaum showed that on average, women under stress have less activation of the HPA-axis compared to men and thus have lower levels of inflammation [39]. Furthermore, Taylor and colleagues reported that women are more likely to seek out social support in response to social stress, whereas men tend to exhibit a fight-or-flight stress response [40]. Different biological responses to social stress between men and women may contribute to the lack of association between cancer mortality and inflammation found in our female cohort.

While we found social isolation to be correlated with multiple potential risk factors for cancer, including heightened fibrinogen levels, increased obesity and smoking levels, and low physical activity and socioeconomic status, we found no association between social isolation and rate of cancer mortality. In contrast to our findings, Reynolds and Kaplan found higher rates of cancer mortality in socially isolated women after adjusting for age, smoking, physical health, household income, and alcohol consumption [41]. Although their model adjusted for age at cancer diagnosis and stage of disease, the authors did not account for inflammation and physical activity as presented in this current report. Furthermore, in a meta-analysis of 40 observational studies, Leigh-Hunt et al. found that social isolation was correlated with poor cardiovascular and mental health outcomes, but not cancer mortality [42].

In this report, we studied social isolation specifically in women rather than among all adults. Previous studies have found that social isolation may affect men and women differently. In one report, Yang et al. found that socially isolated men had an increase in cancer mortality, but not isolated women [6]. This study of 3082 men found that socially isolated men had a 62% higher cancer mortality rate compared to those who were not isolated, whereas there was no relationship between social isolation and cancer mortality among women. On the other hand, Marcus et al. found that socially isolated women had a 39% higher cancer mortality rate compared to women who were not isolated after adjusting for age, sex, race-ethnicity, and socioeconomic status. Although the authors used a more comprehensive dataset composed of NHANES 1988–1994 and the 1990 Census, the authors did not adjust for chronic illnesses, NSAID use, inflammation, or other health measurements such as BMI and physical activity [11]. After adjusting for the aforementioned factors, our study found that social isolation was associated with inflammation, but not with cancer mortality.

Our results may not be generalizable due to the following limitations. Our sample was restricted to 1988–1994 due to the limited span of Social Network Index scores collected by NHANES. Due to the cross-sectional nature of our study, we were unable to offer insight into how changes over time in factors such as social isolation and inflammation impacted our participants’ health outcomes. Furthermore, our study did not exclude women who had a prior history of chronic diseases other than cancer; studies have shown that diseases such as diabetes, cardiovascular disease, auto-immune disease, and hepatic and renal disease are associated with chronic inflammation and may have confounded our results [43]. We were also unable to obtain information on cancer incidence, tumor type, stage of disease, treatment, and survival time after diagnosis. The incidence and outcomes of certain cancers may be related to inflammation, while others may be predominantly related to other causes. For example, incidence and outcomes are largely related to carcinogens in smoking-related cancers. By including all cancer types, we may not be able to detect associations between certain subsets of malignancy and inflammation. Moreover, the surveys did not focus on factors related to social isolation such as feelings of loneliness, stress, or depression, which may affect cancer mortality. However, our report is a cross-disciplinary study that relates social behavior, immunology, and oncology. Furthermore, we were able to perform our analysis in a multivariate model adjusting for important demographic, health, and behavioral factors.

## Conclusions

This is one of few studies to evaluate the role of social isolation on inflammation and cancer mortality in women. We found that social isolation was associated with increased inflammation, in particular fibrinogen. However, our results did not find an association with social isolation and cancer mortality after adjusting for race, socioeconomic status, body mass index, comorbidities, and substance use. Novel inflammatory biomarkers and metrics for social isolation may further enhance our understanding of the relationship among social disconnectedness, inflammation, and cancer mortality.

## Supplementary Information

**Additional file 1: Supplemental Table 1.** Multivariate Cox Proportional Hazard Model of Associated Factors Including Amount of Smoking and Alcohol Consumption with Cancer Mortality.

## Data Availability

The datasets generated and/or analyzed during the current study are publicly available in the NHANES repository, (https://wwwn.cdc.gov/nchs/nhanes/Default.aspx).

## Abbreviations

CRP: C-reactive protein
BMI: Body mass index
NHANES: National Health and Nutrition Examination Survey
MEC: Mobile Examination Center
PIR: Poverty income ratio
SNI: Social Network Index
NDI: National Death Index
IRB: Institutional Review Board
HR: Hazard ratio
CI: Confidence interval
HPA: Hypothalamic Pituitary Adrenocortical
