# Supplementary material for: Social isolation, inflammation, and cancer mortality from the National Health and Nutrition Examination Survey - a study of 3,360 women
Source: BMC Public Health. 2021 Jul 2;21:1289. doi: 10.1186/s12889-021-11352-0 (PMC8252233; doi:10.1186/s12889-021-11352-0)
Supplement: Supplementary file 1 — Additional file 1: Supplemental Table 1. Multivariate Cox Proportional Hazard Model of Associated Factors Including Amount of Smoking and Alcohol Consumption with Cancer Mortality. [file 12889_2021_11352_MOESM1_ESM.docx]

Supplemental Table 1. Multivariate Cox Proportional Hazard Model of Associated Factors Including Amount of Smoking and Alcohol Consumption with Cancer Mortality

| **Characteristics** | **Hazard Ratio** | **95% Confidence Interval** | **P-value** |
| --- | --- | --- | --- |
| ***Social Network Index*** |  |  |  |
| 4=Not isolated | 1 |  |  |
| 3=Somewhat isolated | 0.70 | 0.40-1.24 | 0.22 |
| 2=Very isolated | 0.79 | 0.38-1.64 | 0.52 |
| 0/1=Most isolated | 0.72 | 0.26-2.01 | 0.53 |
| ***Race/Ethnicity*** |  |  |  |
| White | 1 |  |  |
| Black | 1.40 | 0.85-1.29 | 0.18 |
| Mexican American | 1.16 | 0.70-1.91 | 0.57 |
| Other^a^ | 1.11 | 0.23-5.49 | 0.89 |
| ***Income Level*** |  |  |  |
| Low income | 1 |  |  |
| Middle income | 0.89 | 0.57-1.39 | 0.6 |
| High income | 1.02 | 0.63-1.65 | 0.93 |
| ***Education*** |  |  |  |
| Fewer than 12 years | 1 |  |  |
| 12 years or more | 1.38 | 1.01-1.89 | 0.04 |
| ***BMI (kg/m^2^)*** |  |  |  |
| <30.0 kg/m^2^ | 1 |  |  |
| ≥30.0 kg/m^2^ | 1.53 | 1.07-2.18 | 0.02 |
| ***Diabetes Diagnosis*** |  |  |  |
| No | 1 |  |  |
| Yes | 0.72 | 0.34-1.55 | 0.40 |
| ***Cardiovascular Disease Diagnosis*** |  |  |  |
| No | 1 |  |  |
| Yes | 0.78 | 0.49-1.24 | 0.29 |
| ***Smoking Status*** |  |  |  |
| Nonsmoker | 1 |  |  |
| Former | 0.84 | 0.57-1.22 | 0.35 |
| Current |  |  |  |
| <1 pack/day | 1.40 | 0.90-2.15 | 0.13 |
| ≥1 pack/day | 2.14 | 1.50-3.04 | <0.001 |
| ***Alcohol Consumption*** |  |  |  |
| Nondrinker | 1 |  |  |
| Former | 1.04 | 0.80-1.36 | 0.77 |
| Current |  |  |  |
| 1-2 drinks/day | 0.78 | 0.55-1.11 | 0.16 |
| >2 drinks/day | 1.36 | 0.88-2.11 | 0.16 |
| ***Relative Physical Activity*** |  |  |  |
| About the Same | 1 |  |  |
| Less Active | 1.54 | 1.06-2.23 | 0.02 |
| Most Active | 0.67 | 0.52-0.87 | 0.004 |
| ***Self-Reported Health Status*** |  |  |  |
| Excellent | 1 |  |  |
| Very good | 1.49 | 1.001-2.22 | 0.049 |
| Good | 1.36 | 0.96-1.93 | 0.08 |
| Fair | 1.06 | 0.63-1.78 | 0.82 |
| Poor | 0.41 | 0.14-1.16 | 0.09 |
| ***Non-Steroidal Anti-Inflammatory Drug Use*** |  |  |  |
| Nonuser^b^ | 1 |  |  |
| User | 1.12 | 0.80-1.57 | 0.50 |
| ***C-reactive Protein (mg/dL)*** |  |  |  |
| ≤0.21 mg/dL | 1 |  |  |
| 0.22-0.63 mg/dL | 1.18 | 0.80-1.74 | 0.40 |
| >0.63 mg/dL | 0.86 | 0.52-1.42 | 0.55 |
| ***Fibrinogen (mg/dL)*** | 1.001 | 0.998-1.003 | 0.57 |

^a^ Other race consists of mixed race, Asians, and any other race not specified in the NHANES III database
^b^ Non-steroidal anti-inflammatory drugs (NSAID) nonuser includes those who never taken NSAID and never taken NSAID on a regular basis (<30 pills/month)
